# A new Multi Locus Variable Number of Tandem Repeat Analysis Scheme for epidemiological surveillance of *Xanthomonas vasicola* pv. *musacearum*, the plant pathogen causing bacterial wilt on banana and enset

**DOI:** 10.1371/journal.pone.0215090

**Published:** 2019-04-11

**Authors:** Gloria Valentine Nakato, Juan Luis Fuentes Rojas, Christian Verniere, Laurence Blondin, Teresa Coutinho, George Mahuku, Emmanuel Wicker

**Affiliations:** 1 IITA, Kampala, Uganda; 2 Department of Biochemistry, Genetics and Microbiology, Centre for Microbial Ecology and Genomics/Forestry and Agricultural Biotechnology Institute, University of Pretoria, Pretoria, South Africa; 3 UMR IPME, Univ Montpellier, CIRAD, IRD, Montpellier, France; 4 CIRAD, UMR BGPI, Montpellier, France; 5 IITA, Dar Es Salaam, Tanzania; 6 CIRAD, UMR IPME, Montpellier, France; National Cheng Kung University, TAIWAN

## Abstract

*Xanthomonas vasicola* pv. *musacearum* (Xvm) which causes *Xanthomonas* wilt (XW) on banana (*Musa accuminata* x *balbisiana*) and enset (*Ensete ventricosum*), is closely related to the species *Xanthomonas vasicola* that contains the pathovars *vasculorum* (Xvv) and *holcicola* (Xvh), respectively pathogenic to sugarcane and sorghum. Xvm is considered a monomorphic bacterium whose intra-pathovar diversity remains poorly understood. With the sudden emergence of Xvm within east and central Africa coupled with the unknown origin of one of the two sublineages suggested for Xvm, attention has shifted to adapting technologies that focus on identifying the origin and distribution of the genetic diversity within this pathogen. Although microbiological and conventional molecular diagnostics have been useful in pathogen identification. Recent advances have ushered in an era of genomic epidemiology that aids in characterizing monomorphic pathogens. To unravel the origin and pathways of the recent emergence of XW in Eastern and Central Africa, there was a need for a genotyping tool adapted for molecular epidemiology. Multi-Locus Variable Number of Tandem Repeat Analysis (MLVA) is able to resolve the evolutionary patterns and invasion routes of a pathogen. In this study, we identified microsatellite loci from nine published Xvm genome sequences. Of the 36 detected microsatellite loci, 21 were selected for primer design and 19 determined to be highly typeable, specific, reproducible and polymorphic with two- to four- alleles per locus on a sub-collection. The 19 markers were multiplexed and applied to genotype 335 Xvm strains isolated from seven countries over several years. The microsatellite markers grouped the Xvm collection into three clusters; with two similar to the SNP-based sublineages 1 and 2 and a new cluster 3, revealing an unknown diversity in Ethiopia. Five of the 19 markers had alleles present in both Xvm and *Xanthomonas vasicola* pathovars *holcicola* and *vasculorum*, supporting the phylogenetic closeliness of these three pathovars. Thank to the public availability of the haplotypes on the MLVABank database, this highly reliable and polymorphic genotyping tool can be further used in a transnational surveillance network to monitor the spread and evolution of XW throughout Africa.. It will inform and guide management of Xvm both in banana-based and enset-based cropping systems. Due to the suitability of MLVA-19 markers for population genetic analyses, this genotyping tool will also be used in future microevolution studies.

## Introduction

Plant disease emergences constitute a major threat for global food security worldwide. These emergences may gain in importance due to the increase of plant transnational exchanges, crop intensification, and climate change [1]. For better prevention and control of these emerging diseases, it is essential to understand the origin of plant pathogens, decipher the evolutionary mechanisms leading to pathogen adaptation to new crops, and identify the ecological factors (including the crop system composition) favoring this adaptation.

Understanding how the pathogen genetic diversity is distributed and structured over time and space, can allow to infer migration patterns, identify source populations, estimate the efficient population size. Thus, assessing the pathogen genetic diversity and structure can give access to the driving forces of pathogen population dynamics. Moreover, an unbiased assessment of the pathogen genetic diversity is a prerequisite for building up reliable pathogen-informed management strategies, notably involving crop resistance. The exploration of these questions requires the implementation of a molecular epidemiology approach, including properly sampled bacterial collections and adequate genotyping tools.

From the early 2000s, an outbreak of *Xanthomonas* wilt disease caused by the economically important *Xanthomonas vasicola* pv. *musacearum* (Xvm, synonym *Xanthomonas campestris* pv. *musacearum*) threatened banana (*Musa accuminata* x *balbisiana*) and enset (*Ensete ventricosum*) production within the East and Central African (ECA) countries [2]. The disease affects all cultivated types of both crops, although damaging more severely the ABB type in banana [3, 4]. Despite its economic impact on banana and enset production, little is known about the population biology and epidemiology of Xvm. Several comparative genomics studies revealed the phylogenetic closeness of Xvm to the *vasculorum* and *holcicola* pathovars of *Xanthomonas vasicola* [5, 6], supporting a reclassification of the originally described *Xanthomonas campestris* pv. *musacearum* into the species *X*. *vasicola*. We will thus refer to the pathogen as *Xanthomonas vasicola* pv. *musacearum* (Xvm) in this research. Molecular typing tools including Rep-PCR, random amplification of polymorphic DNAs (RAPDs), enterobacterial repetitive intergenic consensus-PCR (ERIC-PCR) and single-nucleotide polymorphisms (SNPs) were developed to elucidate genetic relationships among Xvm populations [2, 5, 6]. However, due to the low inter- or intra-laboratory reproducibility for the first two methods and to the low sequence diversity contained in Xvm, such markers have been unable to resolve the Xvm evolutionary patterns and invasion routes [7]. SNP typing revealed four haplotypes within the Xvm population from east Africa and Ethiopia, and low levels of polymorphism outside Ethiopia [8]; as an example, populations sampled 10 years apart (2005 and 2014) in Uganda shared the same unique haplotype [8]. With these results, discriminative molecular typing tools for population studies such as Multi-Locus Variable Number of Tandem Repeat Analysis (MLVA) are needed. Such markers have the power to discriminate between closely related strains providing a better understanding of outbreaks and tracing back the pathways of spread from inoculum sources.

MLVA is based on the detection of tandem repeat (TR) polymorphisms within the genome of an organism [9, 10]. Tandem repeats (TR) with short repetitive DNA sequences, called microsatellites when smaller than a 9 bp unit, are highly variable within bacterial genomes [11, 12] and may resolve the genetic diversity of monomorphic pathogens [7, 13]. Variability in the number of TRs is mainly generated through slipped strand mispairing during DNA replication [14]. Short TR loci mainly evolve following either a stepwise mutation model (SMM) where new alleles are created by the addition or deletion of a single repeat unit [15] or a generalized two-phase model [16] where the distribution of the numbers of multiple-repeat mutations approximate a geometric distribution [17]. The mutation rates differ between TR loci within a wide range as a result of TR locus specific properties [18]. A high mutation rate increases the likelihood of size homoplasy of TR alleles, i.e. they are identical by state (a same allele size) but have different identity by descent. Size homoplasy may distort the information provided by highly variable loci but this distortion can be minimized by increasing the number of loci and combining markers with different genetic diversity values [16, 19].

MLVAs were first used to characterize highly monomorphic bacterial pathogens within the medical field [20–22], and have now been successfully used to evaluate diversity in plant pathogens such as *Ralstonia solanacearum* [23–25], *Xanthomonas citri* subsp. *citri* [26, 27], *Xanthomonas oryzae* pathovars [28, 29], *Xylella fastidiosa* [30], *Xanthomonas axonopodis* pv. *manihotis* (Xam) [31], *Clavibacter michiganesis* [32] and *Erwinia amylovora* [33]. The advantages of MLVAs include ease of performance, high reproducibility and discriminatory power, portability and rapidity, as well as tremendous reduction in cost since they can be amplified directly from a bacterial colony without need for DNA extraction [31, 34, 35]. In addition, MLVAs allow for analysis of larger numbers of samples and loci due to reduction in sequencing costs [36, 37].

In this study, we describe the development of a new genotyping method for Xvm, based on MLVA. The MLVA scheme was evaluated on a collection of 335 Xvm strains from five countries, and its discriminatory power was compared to the SNP-derived typing method. This work demonstrates the usefulness and power of the MLVA scheme targeting 19 TR loci for monitoring Xvm populations and epidemics at different temporal and geographical scales.

## Materials and methods

### Bacterial strains

A total of 335 strains of Xvm from known hosts and potential alternative hosts were collected. They were sampled from Ethiopia (n = 122), Uganda (n = 150) and other parts of Eastern and Central Africa (ECA; namely DR. Congo, Kenya, Rwanda, Tanzania, n = 63) (Table 1). Within this collection, 20 strains were obtained from the National collection of Plant Pathogenic Bacteria (NCPPB) (Table 1). All 335 strains were confirmed to be Xvm using GspDm primers [38] and five new Xvm-specific primers [8]. All bacterial strains were grown on Wilbrink or YPGA medium for 48h at 28°C and stored in W broth/glycerol or YPGA slants at -80°C.

**Table 1:** Summary of the collections of *Xanthomonas vasicola pv*. *musacearum*.

Country	Year	SNP-based haplotype	Strain number
**Part 1**:** Collection genotyped with SNPs and MLVA (n = 63)**			
DRC	2015	Hap_1	3
Ethiopia	1966	Hap_1	1^a^
Ethiopia	1967	Hap_1	1^a^
Ethiopia	2004	Hap_1	1
Ethiopia	2004	Hap_3a	2
Ethiopia	2004	Hap_3b	5
Ethiopia	2004	Hap_4	4
Ethiopia	2015	Hap_3	1
Rwanda	2005	Hap_1	1^a^
Rwanda	2015	Hap_1	4
Tanzania	2015	Hap_2	15
Uganda	2014	Hap_2	25
**Part 2**:** Collection genotyped with MLVA only (n = 272)**			
Burundi	2007	-	1^a^
DRC	2005	-	2^a^
DRC	2015	-	29
Ethiopia	2017	-	103
Ethiopia	2004	-	2
Ethiopia	2015	-	2
Kenya	2006	-	1
Rwanda	2005	-	3^a^
Tanzania	2007	-	4^a^
Uganda	2005	-	6^a^
Uganda	2012	-	78
Uganda	2014	-	6
Uganda	2016	-	35

^a^ Strain obtained from the National Collection of Plant Pathogenic Bacteria (NCPPB).

Furthermore, the specificity of the MLVA scheme was assessed on phylogenetically close strains. Since Xvm genomes are most similar to members of the species *X*. *vasicola*, we tested a collection of reference strains of *X*. *vasicola* pv. *vasculorum* (n = 6), *X*. *vasicola* pv. *holcicola* (n = 5), originating from Africa, Reunion Island, and USA (DNA kindly given by J. Lang—Colorado State University) (Table 2). Other *Xanthomonas* species, phylogenetically closely related to *X*. *vasicola* (Jacques *et al*., 2016) were tested: *Xanthomonas oryzae* pv. *oryzae* (n = 2), *Xanthomonas campestris* pv. *cannabis* (n = 1), and *Xanthomonas citri* pv. *citri* (n = 1).

**Table 2:** Core-collection of *Xanthomonas vasicola pv*. *musacearum* and *Xanthomonas vasicola* strains.

Code	CFBP	NCPPB	Species	Host	Country	Location	Year of isolation	Genome^a^	Xvm sublineage^d^
**Core-collection *Xanthomonas vasicola pv*. *musacearum***									
CIX 319	CFBP7123	NCPPB2005	*X*. *vasicola* pv. *musacearum*	*Ensete ventricosum*	Ethiopia	NA	1966	AKBE00000000.1^b^	I
CIX 318	CFBP7122	NCPPB2251	*X*. *vasicola* pv. *musacearum*	*Musa* sp.	Ethiopia	NA	1967	LGTY00000000.1^b^	I
CIX 308	CFBP7166	NCPPB4378	*X*. *vasicola* pv. *musacearum*	*Musa* sp.	Uganda	NA	2005	NA	Unknown
CIX 309	CFBP7167	NCPPB4386	*X*. *vasicola* pv. *musacearum*	*Musa* sp.	Uganda	NA	2005	NA	Unknown
Xvm 4387	CFBP7168	NCPPB4387	*X*. *vasicola* pv. *musacearum*	*Musa* sp.	DR. Congo	NA	2005	SRR494494.1^b^	I
CIX 310	CFBP7169	NCPPB4388	*X*. *vasicola* pv. *musacearum*	*Musa* sp.	DR. Congo	NA	2005	NA	Unknown
CIX 311	CFBP7170	NCPPB4389	*X*. *vasicola* pv. *musacearum*	*Musa* sp.	Rwanda	NA	2005	SRR494495.2^b^	I
CIX 312	CFBP7171	NCPPB4390	*X*. *vasicola* pv. *musacearum*	*Musa* sp.	Rwanda	NA	2005	NA	Unknown
CIX 317	CFBP7172	NCPPB4391	*X*. *vasicola* pv. *musacearum*	*Musa* sp.	Rwanda	NA	2005	NA	Unknown
CIX 316	CFBP7173	NCPPB4392	*X*. *vasicola* pv. *musacearum*	*Musa* sp.	Tanzania	NA	2007	AKBI01000000.1^b^	II
CIX 313	CFBP7174	NCPPB4393	*X*. *vasicola* pv. *musacearum*	*Musa* sp.	Tanzania	NA	2007	NA	Unknown
CIX 314	CFBP7175	NCPPB4394	*X*. *vasicola* pv. *musacearum*	*Musa* sp.	Tanzania	NA	2007	AKBJ00000000.1^b^	II
CIX 315	CFBP7176	NCPPB4395	*X*. *vasicola* pv. *musacearum*	*Musa* sp.	Tanzania	NA	2007	SRR494490.2^b^	II
Xvm 4433	NA	NCPPB4433	*X*. *vasicola* pv. *musacearum*	*Musa* sp.	Burundi	NA	2007	SRR494496.1^b^	II
Xvm 4434	NA	NCPPB4434	*X*. *vasicola* pv. *musacearum*	*Musa* sp.	Kenya	NA	2006	SRR494497.1^b^	II
**Collection *X*. *vasicola***									
19	NA	NCPPB 1241	*X*. *vasicola* pv. *holcicola*	*Sorghum vulgare*	Australia	NA	1962	NA	
423	NA	NA	*X*. *vasicola* pv. *holcicola*	*Sorghum bicolor*	South Africa	NA		NA	
453	NA	NA	*X*. *vasicola* pv. *holcicola*		USA	Nebraska		NA	
CFBP 2543	CFBP 2543	NCPPB 2417	*X*. *vasicola* pv. *holcicola*	*Sorghum vulgare*	New Zealand	NA	1969	JSBW00000000.2^c^	
989	NA	NCPPB 989	*X*. *vasicola* pv. *holcicola*	*Holcus* sp.	USA	Kansas	1961	NA	
454	NA	NA	*X*. *vasicola* pv.*vasculorum*	*Saccharum officarum*	Mauritius	NA		NA	
387	NA	NA	*X*. *vasicola* pv.*vasculorum*	*Zea mays*	USA	Nebraska		NA	
444	NA	NA	*X*. *vasicola* pv.*vasculorum*	*Zea mays*	USA	Kansas		NA	
459	NA	NA	*X*. *vasicola* pv.*vasculorum*	*Zea mays*	USA	Colorado		NA	
Xvv 1381	NA	NCPPB 1381	*X*. *vasicola* pv.*vasculorum*	*Saccharum officinarum*	Zimbabwe	NA	1962	AKBL00000000.1^c^	
Xvv 0206	CFBP 7162	NCPPB 206	*X*. *vasicola* pv. *vasculorum*	*Zea mays*	South Africa	NA	1948	AKBM00000000.1^c^	

^a^ Genome identified by GenBank accession number, or SRA accession number. NA: not available.

^b^ Source: Wasukira *et al* 2012.

^c^ Source: Sapp M. and Studholme D, unpublished.

^d^ Xvm sublineages (SL) as named by Wasukira *et al*. (2012).

### DNA extraction

Strains were first grown on a carbon source-free agar medium (Yeast extract-Peptone-Agar) at 28°C for 24h, and DNA extracted using the Wizard Genomic DNA Purification kit (Promega, Charbonnières-les-Bains, France). DNA quantification and quality control were performed using a Tecan Infinite 200 NanoQuant microplate reader (Tecan Trading AG, Switzerland); DNA was diluted to a final concentration of 10 ng.μL^−1^ using milliQ water, and stored at −20 °C until use.

### *In silico* identification of MLVA loci

Nine Xvm genomes (NCPPB 2005, 2251, 4379, 4380, 4381, 4384, 4392, 4394, and 4434) from the National Centre for Biotechnology Information (NCBI) public database were screened for VNTR loci using the online tool ‘Polloc-V’ (http://bioinfo-web.mpl.ird.fr/xantho/utils/) developed by Luis-Miguel Rodriguez-R and Ralf Koebnik. We used Tandem Repeat Finder, TRF [39] as a TR detection algorithm proposed with the following selection criteria: full loci size from 50 to 400 bp; pattern size from 5 to 9 bp; number of repetitions set to 6 or above; stringency set to maximal values 2 (match) -7 (mismatch) -7 (indel); minimum percentage of similarity between repeat units: 80%. In prokaryotic genomes, short tandem repeats (1–4 nt) are very rare in large genomes with high GC-content [40]. A preliminary screen gave no results for pattern size below 5, so we further focused on larger motifs.

Polloc-V identifies groups of loci based on the similarity rate of their flanking regions. After visual inspection of the 36 loci groups identified, 32 groups were further retained based on the criteria that: each was present in a single locus per genome, with a locus of the same sequence. For each identified locus, the repetition sequences of the motif and the 500 bp-upstream and downstream flanking regions were transferred and concatenated using Geneious v.9.2 [41], to design PCR primers.

### Definition and selection of the PCR primers

PCR primers (20- to 27- nucleotides long) were designed in the flanking regions of the tandem repeat sequence using Geneious v. 9.2. Melting temperatures were set around 68 °C to allow downstream primer multiplexing with the QIAGEN Multiplex kit. Primer design parameters were set to be stringent, to avoid the formation of primer dimers and hairpins and to allow downstream multiplexing, and the annealing temperature parameter was set at Melting Temperature minus 4°C (Tm—4°C).

Using BLASTN [42] under Geneious, primers and their corresponding TRs were searched for within the nine Xvm reference genomes to determine the location of each locus (inter- or intragenic), and to verify that (i) each motif corresponded to a single locus per genome, and (ii) the locus-corresponding primers exactly targeted the genomic region containing the locus. Only primers fulfilling these criteria were selected for subsequent analyses. Hence, 21 loci and their corresponding primers were selected for development of the MLVA scheme.

### Preliminary PCR screening

This first screening assessed typeability (ability to amplify all strains of a given lineage or species), reproducibility, and polymorphism of the identified loci. These criteria were assessed on a core-collection comprising of 15 Xvm strains from Burundi, DR. Congo, Ethiopia, Kenya, Rwanda, Tanzania and Uganda representing the geographical distribution of the pathogen.

PCR amplifications were done using the Multiplex PCR kit (Qiagen) in a total volume of 15μL. PCR cycles consisted of one initial denaturation (95°C for 15 minutes) to activate the « hot start » Polymerase, followed by 25 cycles of denaturation at 94°C for 30s, annealing (55°C to 62°C) for 90s, elongation at 72°C for 90 s; and a final elongation step at 60°C for 30 minutes. Electrophoresis of PCR products was done on 3% agarose gel at 100V for 45 minutes. From this screening, 19 polymorphic loci and primer pairs were retained for the following steps.

### Primer multiplexing

The multiplexing consisted of three to four loci mixes per PCR reaction. Each of the multiplex PCR reactions was optimized by testing three hybridization temperatures (57, 60 and 63°C) per mix, according to QIAGEN indications, on seven bacterial strains of the core Xvm collection. The optimal hybridization temperature was determined by visualization of amplicon intensity on 3% agarose gel electrophoresis. The combinations of the different loci in each reaction mix were chosen according to the size ranges of the PCR products, in order to avoid overlapping fragment sizes.

### Genotyping on ABI3500 capillary sequencer

The "forward" primers of each pair were labeled with a fluorophore: 6-FAM, blue (Eurogentec, Angers, France); VIC, green; NED, black; or PET, red (Applied Biosystems, Life Technologies, Saint Aubin, France). The labeling of the different primers was chosen according to the size and intensity of each PCR product: the NED and PET fluorophores being assigned to the smaller fragments, 6-FAM to the primers giving fragments of weak intensity, and VIC to primers giving larger fragments.

Pools of four pairs of labeled primers corresponding to each locus were established (Table 3) and tested in multiplex PCR, using the Multiplex PCR kit (QIAGEN, Courtaboeuf, France) according to manufacturer recommendations. Reaction mixtures (15 μL) consisted of 0.2 μM of each primer (forward primer labelled with one of the fluorescent dyes 6-carboxyfluorescein FAM, NED, PET, and VIC, 2X QIAGEN Multiplex MasterMix, 5X Q-solution and 2 μL of bacterial genomic DNA (10 ng.μL^−1^). PCR reactions consisted of an initial denaturation step of 15 min at 95 °C; 25 cycles of 30 s at 94 °C, at annealing temperatures of either 60 or 63°C, 90 s at 72 °C, and a final 30 min step at 60 °C. Each PCR product was diluted to 100^−1^ and 1.5 μL of diluted PCR product was added to 1.5 μL Hi-Di Formamide (for GeneScan -500 LIZ) and 12 μl GeneScan -500 LIZ internal size standard (Applied Biosystems). The 100-fold dilution was chosen following preliminary tests of different PCR loading volumes, because it gave peaks of good intensity (3000–10000 fluorescence units) with no stutter peaks and rare fluorescence saturation phenomena. This was done to avoid peaks saturating the electropherogram and enable accurate analysis. Capillary electrophoresis was conducted on the ABI3500XL DNA Analyzer 24-channel sequencer (Applied Biosystems).

**Table 3:** Microsatellites, primer description and amplification conditions of the MLVA-19 scheme.

Locus	Primer	Sequence (5’– 3’)	Genome of origin	Tandem repeat sequence	Product Size	Multiplex mix	Tan (°C)^a^
XVM022	XVM022_289F	TGTCCGCATATCCAGCACGC	NCPPB4379	GGCTGCT	313	MIX01	60
	XVM022_601R	GATATCCCAGCCGCACGTCTTG					
XVM002	XVM002_353 F	CCAGGCCACCACGTAATTCAGTCAGG	NCPPB4379	TCGCTG	236		
	XVM002_588 R	CGAACTGCAAAAGCCAAGCCAGAG					
XVM028	XVM028_459F	TGAGGGCAACTAGATCGACGGGTTC	NCPPB4379	GGGAATC	200		
	XVM028_658R	CAGACGGATTTGTTCAACGCATCGCAT					
XVM029	XVM029_310F	CGATATTGGGGTTCTGGCTAGGGTC	NCPPB2005	TTGCAC	375		
	XVM029_684R	AAGTGACGTTTGAGGGGCGC					
XVM020	XVM020_450F	CGTTATTGATCTGACGTATTGCCCATCG	NCPPB4380	GACGCAC	365	MIX02	60
	XVM020_814R	ACTTCATGCCACCCACGTTGC					
XVM030	XVM030_333 F	TGGTGGATGGATGGGTGTTGGTGGT	NCPPB2005	TTGTTGC	250		
	XVM030_582 R	CCCCGGAGAAGCAAGAACCTAGAACCT					
XVM027	XVM027_367 F	GCTCCCGATCCAACGCTTGCTCATG	NCPPB4379	AGAGCCG	228		
	XVM027_594 R	CGCTGCTCCTGGTTCAATTTCCCGATT					
XVM015	XVM015_460F	CGACCAGACCGCCTTGTTCAGAGAAAT	NCPPB4379	AGCGCACGG	249		
	XVM015_708R	GGGATGGTGTTGCTGATGTGGTTTTGC					
XVM016	XVM016_295F	ACTTCTCCACGCCTCTGTTTGCC	NCPPB4384	GGCTATT	377	MIX03	63
	XVM016_671R	GATCTTAACGCTTCCTTGACATCGGC					
XVM021	XVM021_454F	GTCGTTGAAGCGTTCCATGAAGCCG	NCPPB4379	CTTCTGCG	238		
	XVM021_691R	TGTCCTTGGATGAACAAAAGCCCTCGA					
XVM035	XVM035_419F	TTGAATCCAACGGTGCCCTGTCC	NCPPB4380	GCACCAA	225		
	XVM035_643R	GTGCCATGTGTTTCCCCTAGTGTGC					
XVM024	XVM024_387F	CGATCCCAACTCGCCGATGA	NCPPB4379	GCATCGT	323		
	XVM024_709R	CGTACTTCAAGATCACCGCAGAGCAT					
XVM006	XVM006_107 F	GGTAGCGGTGTGGGTTGCGAAGAC	NCPPB4379	GGGATTC	427	MIX04	63
	XVM006_533 R	GGCTACGAGGTGGATGTGCAGGTG					
XVM018	XVM018_440F	GAACTGCTGTAACCGTCGATTGCCTC	NCPPB4434	TGAGTGC	269		
	XVM018_708R	GCGTCACCTACTCCGTTGCCAGAT					
XVM038	XVM038_404F	ACGGTAGTAATGGGCAGCAGGGTG	NCPPB4379	CGGTGGTGGCTT	227		
	XVM038_630R	CGGTGTCGTTCGAGAAGCTCAAGATAGA					
XVM014	XVM014_402F	AGGTTCCAGGTCACGCAGATTCTTGT	NCPPB4384	GAATTGG	316		
	XVM014_718R	GACTGGTGTGGATGGGCGTTCT					
XVM005	XVM005_376F	AAGCAGCCACGGAAAGGACAGG	NCPPB2005	CGCCAG	362	MIX05	63
	XVM005_737R	TGACCACTGCCGCACACCAA					
XVM036	XVM036_164 F	CTGGCTGCTCAAGGACATCACCAACC	NCPPB4384	TCCCGAA	386		
	XVM036_549 R	GGTAGCGGTGTGGGTTGCGAAGA					
XVM012	XVM012_223F	TGCGTACCGAACTCTGTGGCTAC	NCPPB2005	TGGCGG	376		
	XVM012_598R	GGTTGTGCGAACTTTTGTCGTGCTAC					

^a^ T_an_: annealing temperature.

Analyses were conducted at the GenSeq technical facilities of the « Institut des Sciences de l’Evolution de Montpellier »—Labex CEMEB “Centre Méditerranéen de l’Environnement et de la Biodiversité”.

### Analysis of a core-collection using SNP-derived RFLP markers

A collection of 63 strains was analyzed using the MLVA-19 scheme and SNP-derived RFLP typing tools. Two sets of SNP-derived markers were used. The first set, named WAS-SNPs, targeting 500-bp loci was adopted from Wasukira *et al*. [6] while the second set, named VN-SNPs, targeting between 200 to 600-bp were newly designed to further characterize the Xvm population [8].

### Data analysis

#### Data scoring

Electrophoregramms were analyzed with GeneMapper 4.0 (Applied Biosystems). Peaks were first automatically detected using the analysis panels we defined for each mix. Each peak was then carefully checked by eye, and false peaks (rare artefacts due to fluorescence saturation) were discarded. The reproducibility of the allelic patterns was checked by running several DNA extractions of the strains NCPPB2005 and NCPPB2251, as well as duplicate analyses of eight Ugandan Xvm DNAs. Fragment sizes obtained for each TR locus were transformed to tandem repeats numbers. Subsequently, the allele sizes were transformed into repeat numbers using a home-made script using R version 3.4.0 [43]. The tandem repeat numbers obtained were rounded up to the next integer [12]. All alleles scored by size, and their corresponding “raw” and rounded repeat numbers, are summarized in the S2 Table in Supplemental Information.

#### Analysis of genetic data

The typeability and specificity of each MLVA locus to Xvm, pathovars of *X*. *vasicola*, and other *Xanthomonas* species were evaluated by comparing the percentage of strains amplified.

Principal component analysis (PCA) was performed using the FactoMineR package [44] in R to estimate the contribution of each locus and how they account for the genetic variability described in the current Xvm collection.

We estimated the genotypic resolution of the MLVA scheme [45] and described the genotypic diversity in relation to different combinations of TR loci by a genotype accumulation curve using R (R::poppr:: *genotype_curve* [46]). The curve is generated by sampling x loci randomly and counting the number of multilocus genotypes (MLG) observed. This sampling is repeated r times from 1 to n-1 loci, creating n-1 distributions of observed MLGs [47].

#### Reconstructing evolutionary relationships across Xvm African haplotypes

Haplotype networks were constructed using the algorithm combining global optimal eBURST (goeBURST) and Euclidean distances in the Phyloviz 2 software [48]. It allowed the visualization of the different clonal complexes (groups of haplotypes differing by a single locus, or Single locus variant (SLV)).

The mutation model followed by the MLVA molecular markers was estimated by looking at the locus variation of recently diverging haplotypes, i.e. single-locus variants (SLV) and double-locus variants (DLV), along the haplotype network of the minimum spanning tree. Furthermore, the number of TR repeats involved in the mutation event was examined to determine whether the stepwise mutation model (SMM), i.e. addition or deletion of a single repeat, was supported for these TR loci.

#### Comparison of the discriminatory power and congruence of the typing techniques

MLVA-19 and SNP-derived RFLP typing techniques were compared using the Hunter and Gaston discriminatory Index (HGDI) [49].

Distance matrices calculated from each MLVA-19 and SNP dataset were calculated and compared using the Mantel test performed by the CADM.post function of the R package ape 5.0, with 9,999 permutations [50]. The Mantel correlation coefficients were computed on rank-transformed distance matrices.

#### Genetic structure

The genetic structure of the Xvm population was assessed by Discriminant Analysis of Principal Components (DAPC) using the adegenet package for the R software [51–53] since DAPC is free of any assumption linked to a population genetic model (such as Hardy-Weinberg equilibrium or absence of linkage disequilibrium). The number of clusters was assessed using the function *find*.*clusters*, which runs successive k-means clustering with increasing number of clusters (k) and the optimal number of clusters selected based on lowest Bayesian information criterion (BIC) [52]. Eleven independent k-means and DAPC runs were performed to assess the stability of clustering.

### Deposition to MLVABank website

The MLVA-19 allelic profiles were deposited to the MLVA website dedicated to plant bacterial pathogens http://bioinfo-web.mpl.ird.fr/MLVA_bank/Genotyping/, corresponding to the MLVAbank at http://microbesgenotyping.i2bc.paris-saclay.fr/, to make MLVA-19 data accessible in an interactive way [54]. The website allows viewing databases with sorting and clustering options, submitting queries, and sharing databases which are maintained and managed by different owners once a common agreement is achieved among partners [26].

## Results

### The MLVA scheme MLVA-19 is based on 19 highly polymorphic loci that are evenly distributed on the genome

From the *in silico* screening of VNTR loci and corresponding primers (detailed in Materials and methods), 21 loci were selected that were unique in each genome, and whose corresponding primers were specific to the locus flanking regions.

From the initial screening of a representative Xvm core-collection strains from different countries (n = 15), 19 loci out of the 21 tested were polymorphic, with two to four alleles per locus. Loci XVM013 and XVM023 did not amplify in any strain and were therefore excluded from the downstream analyses. The primers targeting the 19 loci were multiplexed in sets of either four- or three-loci mixes (Table 3). All but one (XVM038) were considered as microsatellite loci, with motif sizes ranging from 6 to 9 nucleotides. The majority (12 of 19) consisted of 7 nucleotide repeats, and most (12 of 19) had an intergenic location (Table 4). Most loci were evenly distributed on the Xvm genome (S1 Fig). The distance between two adjacent loci ranged from 26.9 kb to 1293.7 kbp, except between XVM006 and XVM036 (652 bp), and between XVM030 and XVM002 (1835 bp).

**Table 4:** Nomenclature, location, function and genetic diversity of the 19 TR loci retained in the MLVA scheme of *Xanthomonas vasicola* pv. *musacearum*. The basic statistics^d^ were obtained from the Xvm collection (n = 335).

Locus	Official nomenclature^a^	Location (intra /intergenic)^b^	Coordinates in NCPPB4379 (bp)^c^		Repeat size	Basic statistics^d^		TR numbers range
			Start	End		H_E_	N_A_	
XVM002	NCPPB4379_3.746_6_61_10.2	Hypothetical protein	2152939	2152217	6	0.73	21	6–35
XVM005	NCPPB2005_18.431_6_36_6	intergene	2519193	2518146	6	0.6	7	2–8
XVM006	NCPPB4379_0.145_7_67_9.6	intergene	1288216	1288926	7	0.63	14	7–20
XVM012	NCPPB2005_28.458_6_36_6	intergene	728842	728307	6	0.62	10	2–14
XVM014	NCPPB4384_7.903_7_72_10.3	intergene	69918	70996	7	0.66	13	5–25
XVM015	NCPPB4379_7.618_9_61_6.8	intergene	322772	323832	9	0.66	13	3–27
XVM016	NCPPB4384_1.353_7_53_7.6	intergene	3154338	3155390	7	0.56	7	6–12
XVM018	Kenyan_21.741_7_44_6.3	intergene	512386	513422	7	0.69	6	4–10
XVM020	NCPPB4380_14.211_7_61_8.7	intergene	4481478	4482029	7	0.85	16	5–21
XVM021	NCPPB4379_28.913_8_64_8	Hypothetical protein	4175076	4176139	8	0.82	10	4–13
XVM022	NCPPB4379_30.295_7_55_7.9	Hypothetical protein	4220957	4221499	7	0.64	8	5–13
XVM024	NCPPB4379_20.274_7_90_12.9	intergene	3580348	3579259	7	0.79	11	4–14
XVM027	NCPPB4379_47.799_7_63_9	RNA binding protein	3745391	3746453	7	0.92	25	7–32
XVM028	NCPPB4379_7.722_7_57_8.1	Hypothetical protein	4397186	4398249	7	0.77	15	5–21
XVM029	NCPPB2005_15.36_6_48_8	Hypothetical protein	3936107	3937148	6	0.61	7	5–13
XVM030	NCPPB2005_4.034_7_42_6	intergene	2151016	2151719	7	0.77	17	6–31
XVM035	NCPPB4380_1.724_7_59_8.4	intergene	3310409	3309372	7	0.56	7	4–10
XVM036	NCPPB4384_0.443_7_67_9.6	intergene	1288274	1288984	7	0.63	16	7–23
XVM038	NCPPB4379_2.022_12.00_86_7.20	Hypothetical protein	4148185	4149270	12	0.5	5	5–9

^a^ Loci were named according to their genome of origin, physical position (kb), TR unit size, total length in the genome of origin, number of repeats [55].

^b^ The intra/intergenic location was assessed on all nine Xvm genomes available.

^c^ The coordinates are based on the Xvm complete genome NCPPB4379 (ASM27789v2, https://www.ncbi.nlm.nih.gov/assembly/GCF_000277895.2), composed of a 4,79 Mb-chromosome and a 49.4 kb-plasmid named pXCM49; all loci were located on the chromosome.

^d^ H_E_, Nei’s gene diversity; N_A_, number of alleles.

Of the 19 loci, 12 contained perfect repeat motifs (no variation of the TR sequence) among which six were interrupted in 3’ (S1 Table). Seven loci contained imperfect repeat motifs (XVM006, XVM014, XVM016, XVM024, XVM029, XVM036, XVM038) with alternative TR sequence displaying only one mismatch from the reference sequence (mostly transitions between Thymine and Cytosin).

Loci XVM027, XVM002, and XVM030 were the most polymorphic, whereas XVM018 and XVM038 were the least polymorphic (Table 4). There was no clear relationship between polymorphism and motif size (S2 Fig), nor with location on the genome, although the least polymorphic locus XVM038 had the greatest motif size (12 bp) and considered a minisatellite.

### The MLVA-19 scheme allowed for good genotypic resolution within Xvm

Contribution of each MLVA marker to the scheme was determined from the Principal Component Analysis (PCA). The first three dimensions explained 66.5% of variance, with axes 1 and 2 being the most informative (respectively 41.9% and 16.88%). Loci XVM030, XVM016, XVM024, XVM022 and XVM020 contributed most to the axis 1, while XVM035, XVM018, XVM012, XVM006 and XVM036 contributed most to axis 2 (S3 Fig); loci XVM002 and XVM028 contributed most to axis 3 (S3 Table, S4 Fig). Some variables were correlated, e.g. XVM006 and XVM036; XVM002 and XVM022; XVM005 and XVM030; XVM015 and XVM038; XVM012 and XVM028 (S3 and S4 Figs).

**Fig 1:**
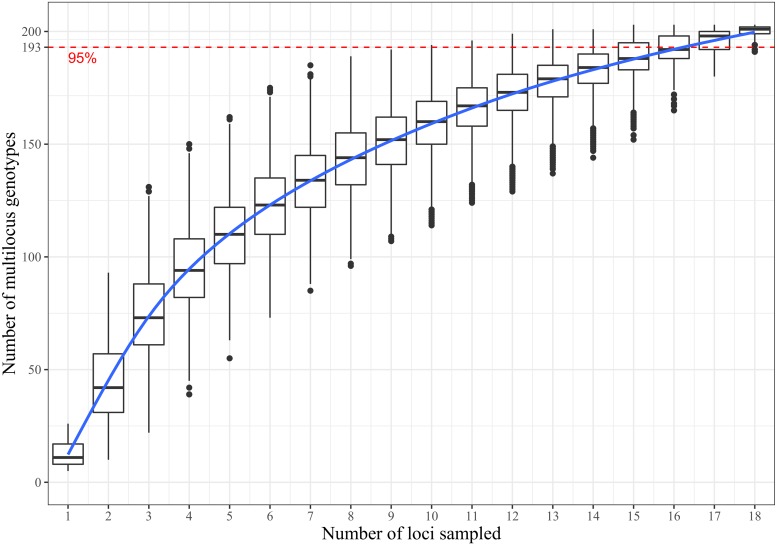
Genotype accumulation curve for 335 strains of *Xanthomonas vasicola pv*. *musacearum* genotyped over 19 loci. The horizontal axis represents the number of loci randomly sampled without replacement up to n-1 loci, the vertical axis shows the number of unique multilocus genotypes observed (n = 208) in the data set. The level of 95% of unique multilocus genotype detected is indicated with a dotted red line.

The genotypic resolution of the MLVA-19 scheme is represented by the genotype accumulation curve (Fig 1). Our set of loci has been shown to be sufficient to accurately resolve the different haplotypes in our sample as the curve reached a plateau with 19 loci. The genotype accumulation curve revealed that more than 90% of the genotypes could be detected with 16 markers, hence the MLVA-19 scheme accurately estimates the clonal diversity of our sample (Fig 1).

### Most of the mutations of our MLVA-19 loci involve single repeat events

In order to estimate the mutation model of the MLVA microsatellite marker evolution, we looked at the variation of repeat numbers between recently diverging haplotypes, i.e. SLVs within the clonal complexes and also with double-locus variants (DLV); most loci were analyzed on more than five evolutionary steps. Thirteen loci (XVM002, 5, 6, 12, 14, 20, 21, 22, 24, 27, 28, 35, and 38) revealed that SLVs resulted from more than 60% of single TR variation, with eight above 80%. XVM012 has a majority (44.44%) of single steps, and XVM014 had 50% of both single and double repeat variation. The locus XVM015 displayed a majority of multiple repeats variations ranging from 2 to 13 repeats. Such events involving large number of repeats could result from recombination mechanism [56]. The loci XVM016, XVM018, XVM029, XVM030 and XVM036 were not involved in SLVs or DLVs. But for those loci, almost all the alleles or number of repeats fulfilled the allelic range observed within our collection (S2 Table).

### MLVA-19 loci are highly typeable on *Xvm* and specific to *X*. *vasicola*

The typeability of most loci was excellent when used on Xvm strains (n = 335), with 18 of the 19 loci amplifying in more than 97% of strains. On the other hand, the locus XVM 015 was amplified in 90.4% of strains (Table 5).

**Table 5:** Typeability and specificity of each XVM locus considering *X*. *vasicola* pv. *musacearum* (Xvm), *X*. *vasicola* pathovars *holcicola* (Xvh), and *vasculorum* (Xvv), and other *Xanthomonas* species: *X*. *oryzae* pv. *oryzae* (Xoo); *X*. *campestris* pv. *cannabis* (Xcc); *X*. *citri* pv. *citri* (Xc), estimated by the number (percentage) of amplified strains.

Locus	Strains amplified within					
	Xvm (n = 335)	Xvv (n = 5)	Xvh (n = 5)	Xoo (n = 2)	Xcc (n = 1)	Xc (n = 1)
XVM002	335 (100)	2 (40)	3 (60)	0 (0)	0 (0)	0 (0)
XVM005	335 (100)	2 (40)	0 (0)	0 (0)	0 (0)	0 (0)
XVM006	327 (97.6)	1 (20)	3 (60)	1 (50)	0 (0)	0 (0)
XVM012	328 (97.9)	0 (0)	0 (0)	0 (0)	0 (0)	0 (0)
XVM014	332 (99.1)	3 (60)	1 (20)	0 (0)	0 (0)	0 (0)
XVM015	303 (90.4)	5 (100)	0 (0)	0 (0)	0 (0)	0 (0)
XVM016	332 (99.1)	4 (80)	2 (40)	0 (0)	0 (0)	0 (0)
XVM018	333 (99.4)	0 (0)	1 (20)	0 (0)	0 (0)	0 (0)
XVM020	333 (99.4)	3 (60)	2 (40)	0 (0)	0 (0)	0 (0)
XVM021	332 (99.1)	2 (40)	1 (20)	0 (0)	0 (0)	0 (0)
XVM022	334 (99.7)	5 (100)	3 (60)	0 (0)	0 (0)	0 (0)
XVM024	335 (100)	5 (100)	0 (0)	0 (0)	0 (0)	0 (0)
XVM027	332 (99.1)	3 (60)	1 (20)	0 (0)	0 (0)	0 (0)
XVM028	335 (100)	3 (60)	2 (40)	0 (0)	0 (0)	0 (0)
XVM029	333 (99.4)	5 (100)	3 (60)	0 (0)	0 (0)	0 (0)
XVM030	334 (99.7)	4 (80)	2 (40)	0 (0)	0 (0)	0 (0)
XVM035	332 (99.1)	4 (80)	0 (0)	0 (0)	0 (0)	0 (0)
XVM036	326 (97.3)	2 (40)	2 (40)	1 (50)	0 (0)	0 (0)
XVM038	333 (99.4)	3 (60)	0 (0)	0 (0)	0 (0)	0 (0)
**Total**	335	5	5	2	1	1

Most of the TR loci of the MLVA scheme for Xvm amplified at least a few strains of *X*. *vasicola* pv. *vasiculorum* (17/19 loci) and pv. *holcicola* (13/19 loci) (Table 5). However, specificity of the loci was variable between Xvm and *X*. *vasicola*. XVM012 was the only Xvm-specific locus and was not amplified in any other *X*. *vasicola* pathovar. Four loci (XVM002, XVM006, XVM022, XVM029) amplified 60% of strains belonging to the pathovar *holcicola* (n = 5), while amplification in *X*. *vasculorum* ranged between 20% and 40%. Seven loci (XVM015, XVM016, XVM022, XVM024, XVM029, XVM030, and XVM035) amplified 40 to 100% of pathovar *vasculorum* strains (n = 5) that were tested (Table 5). Importantly, some loci produced alleles that were both present in Xvm and another pathovar of *X*. *vasicola* (XVM002, XVM005, XVM006, XVM014, XVM015, XVM020, XVM027, XVM028, XVM029, and XVM036). For instance three alleles of the locus XVM006 were shared by Xvm and Xvh, while one allele was shared by Xvm and Xvv (S2 Table, summarizing all alleles by size and repeat numbers).

No locus or very few loci were amplified with other *Xanthomonas* species used in this study.

### MLVA-19 is congruent with the SNP-RFLP based typing method but more discriminative

**Fig 2:**
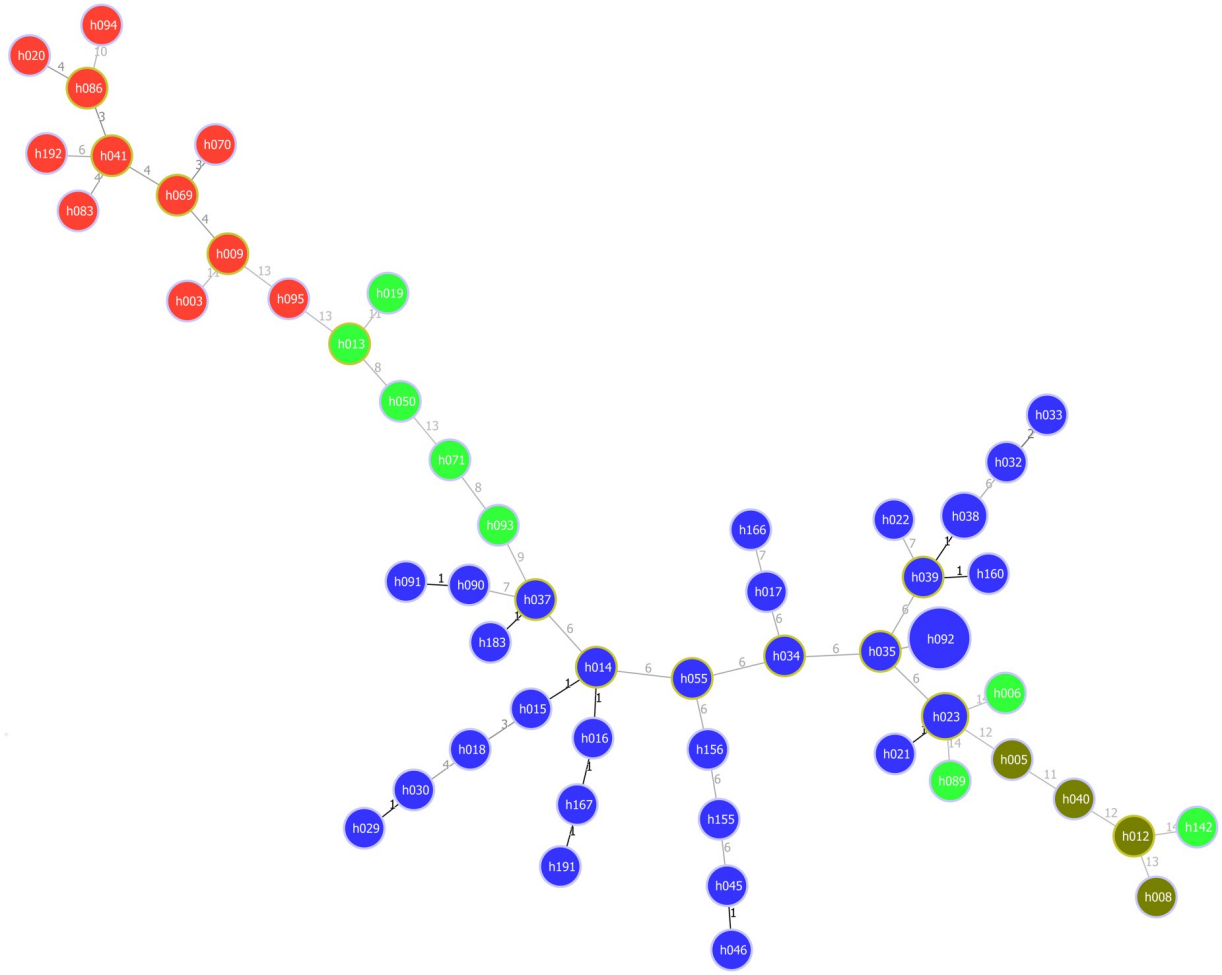
Minimum spanning tree (MST) of *Xanthomonas vasicola pv*. *musacearum* based on SNP haplotypes identified using goeBURST in PHYLOVIZ. Each sequence type (ST) is displayed as a circle with a size proportional to the number of strains by which is represented. The different colors indicate the SNP haplotype. Numbers on the branches indicate the number of locus differences between the neighboring haplotypes.

We estimated the congruence between the MLVA-19 scheme and the SNP-derived typing method from Wasukira *et al*.[6] by three complementary approaches. We first assessed the distribution of SNP sublineages and SNP-based haplotypes on the goeBURST minimum spanning tree drawn from the 53 MLVA-19 haplotypes. Two MLVA clusters were consistent with the SNP-sub-lineages SLI and SLII described by Wasukira *et al*. (Fig 2). Moreover, the MLVA19-haplotypes corresponding to SNP-haplotypes 3 and 4 are also clustered together.

**Fig 3:**
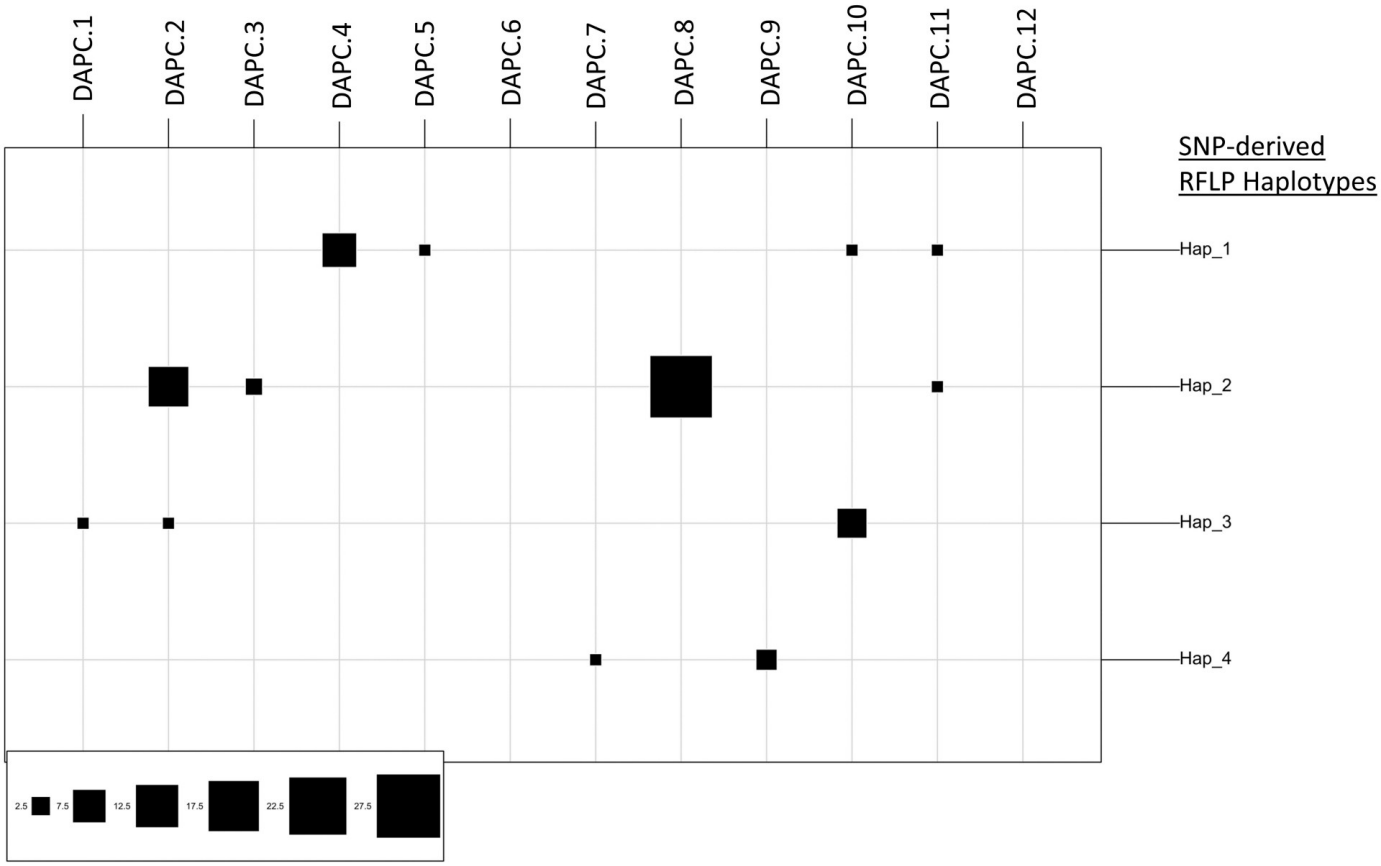
Correspondence between SNP haplotypes with generic clusters inferred by discriminant analysis of principal components. The clusters are shown on the horizontal axis and the countries and SNP haplotypes are indicated on the vertical axis. The black squares represent the number of strains per SNP haplotype within the given cluster. The Figs were created using the table.value function in the R “Adegenet” package [53].

We also assessed whether the 12 genetic clusters identified by DAPC (named DAPC clusters, and described in detail below) based on MLVA-19, corresponded to SNP-based haplotypes [57] (Fig 3). The SLI haplotypes (Hap1) were distributed within clusters 4, 5, 10 and 11, while SLII haplotypes (Hap2) corresponded to clusters 2, 3, 8 and 11; Hap3 and Hapl4 belonged to clusters 1, 2, and 10, and 7 and 9, respectively (Fig 3). Hence, SLI and SLII strains were distributed in distinct clusters, with the exception of cluster 11 (Fig 3). Cluster 11 contained one Hap1-SLI strain, and the SLII strain T35C; but T35C was actually poorly assigned (53%) to cluster 11 while also assigned to SL II-associated cluster 8 (44%). Clusters 2 and 10 were also shared between Hap3 and Hap2 and Hap1, respectively.

We also performed the Mantel tests between distance matrices obtained on a strain subset (n = 32) genotyped by both methods. Distance matrices (Euclidean and Manhattan distances) were highly correlated, with respective Mantel correlation coefficients of 0.412 and 0.492 (P< 0.001 for both Euclidean and Manhattan matrices, 9999 permutations), indicating that genetic distances from MLVA-19 and SNP-based RFLPs were significantly congruent.

A collection of 63 strains was typed with both SNP-derived markers [6, 57] and the MLVA-19 scheme. The discriminatory index was 0.981 for MLVA (n = 63 haplotypes) and 0.564 for the SNPs (n = 6 haplotypes).

### MLVA-19 reveals epidemiological relationships between countries and is discriminative at the field scale

The MLVA-19 scheme distinguished 208 haplotypes among the Xvm collection (n = 335). Twenty-nine clonal complexes (CC) defined as groups of single locus variant (SLV) grouped 51.34% of the strains using the goeBURST algorithm. Numbers of haplotypes per CC ranged from 2 to 11; 13 CC grouped three haplotypes and above. Fifty-five per cent of the haplotypes (n = 114) remained as singletons, differing from each other by four to thirteen loci. Interestingly, no haplotype was shared by strains from different countries. Most CC (n = 26) were country-specific (13 from Uganda, 12 from Ethiopia, one from Tanzania), while three were shared among countries (CC1: Uganda, Tanzania, Burundi, DRC; CC13 and CC29: DRC, Rwanda) (S4 Table).

We also analyzed strains that were isolated from the same field. At this scale, the MLVA-19 scheme discriminated from 8 to 13 haplotypes per field (Ethiopia, n = 2 and Uganda, n = 1) (Table 6). Hence, the MLVA-19 was discriminative enough to distinguish different haplotypes from the country scale to the field scale.

**Table 6:** MLVA-19 is discriminative at the field scale. Number of MLVA-19 haplotypes detected in three fields of Ethiopia and Uganda.

Country and location	Field	Host	No. of strains	No. of haplotypes
ETHIOPIA, Yem	Meleka	Enset	16	13
	Oya Freto	Enset	14	8
UGANDA, Mukono	Kiifu	Banana	10	8

## Discussion

Understanding invasion routes, biology and epidemiology of pathogens is important to elucidate the main factors involved in the invasion process, to develop epidemiological surveillance strategies aimed at preventing new introductions, as well as building pathogen-informed breeding strategies. At small-scales, it is important to determine the source of outbreaks and the means of transmission to limit the pathogen dispersion. In this study, we developed a highly discriminative typing tool that allowed us to elucidate the population structure and diversity of *Xanthomonas vasicola* pv. *musacearum*. The MLVA approach has become a standard in evaluating the population structure and dynamics of bacterial pathogens affecting human, animal and plant health especially due to its ability to detect small yet significant genetic differences.

### MLVA-19 scheme is well suited for molecular epidemiological analysis of *Xanthomonas vasicola* pv. *musacearum*

To our knowledge, MLVA-19 is the first scheme of this type to be developed for Xvm. This scheme consists of 19 loci evenly distributed on the Xvm genome. When choosing markers for analysis, it is important to ensure that the combination of markers selected allow for accurate discrimination of the haplotypes [58]. Principal component analysis and the genotype accumulation curve indicated that these 19 loci are sufficient to accurately discriminate the Xvm population and thus adding more markers would not identify many new genotypes. The genotype accumulation curve assesses the power to discriminate among unique haplotypes given a random combination of loci [47] and resolved almost all the haplotypes with 16 to 17 microsatellite markers, indicating that such a number of loci would be sufficient to detect most of the genetic diversity within Xvm.

MLVA-19 is also well suited for molecular epidemiology analyses. We determined that most loci of the MLVA-19 scheme evolved by gaining or losing a single repeat at one time, supporting a stepwise mutation model. This may facilitate the relatedness analyses and gene genealogies, and makes this MLVA-19-scheme useful in molecular epidemiology study. Several authors have noted that although SMM is considered to be the predominant mutational model for the microsatellites within bacteria, precise data remain scarce on the actual mutation model and possible variations around this model within the *Proteobacteria* phylum [26, 33, 59, 60].

MLVA-19 was also discriminative enough to identify and monitor different haplotypes of Xvm at the field scale, as shown in the banana and enset fields in Uganda, and Ethiopia respectively. This paves the way for future studies addressing the importance of multi-infection events and the spatial dynamics of bacterial infection within and across farm fields, comparing the spatial structures of aerial infestations and soilborne or tool-mediated infestations, among others.

### MLVA-19 are partially transferable to other pathovars of *X*. *vasicola*

All MLVA-19 loci were amplified in Xvm, but several loci amplified within the other pathovars of *X*. *vasicola*. One MLVA scheme initially developed for *Xanthomonas citri* pv. *citri* has also been partly and successfully adapted to a close pathogenic bacterium from the same species [61, 62]. On the other hand, the phylogenetically closest species (*X*. *oryzae*, *X*. *cannabis* [63]) were not amplified by the MLVA-19. Collectively, these findings further provided evidence for a close phylogenetic relatedness between Xvm and other pathovars of *Xanthomonas vasicola*.

### MLVA-19 is congruent with SNP markers, while revealing an unexpected diversity

From our data, the MLVA typing scheme was much more discriminatory than the SNP typing method described by Wasukira et al.[6]. Indeed, SNP and MLVA markers differ in mutation rates and mechanisms with independent evolutionary processes. Although SNP markers are robust phylogenic markers, less prone to distortion via selective pressure, as is the case for repetitive sequences [64]; their mutation rate is much lower than that of TR, and as such, do not offer enough polymorphisms to discover recent evolutionary events. Whereas, MLVAs mutate faster through the addition or subtraction of tandem repeats, producing greater levels of variation and often providing more discriminatory power per marker. Due to their different evolutionary dynamics, MLVAs and SNPs offer complementary information [65]. MLVA is considered suitable for short-term epidemiological analysis, while SNPs are suited to long-term or global epidemiological analyses [66].

MLVA-19 typing confirmed the differentiation of Xvm into two main sublineages as defined by Wasukira et al [6]. Furthermore, MLVA also identified several homogeneous clusters within each of these sublineages with three to four DAPC clusters per sublineage. Interestingly, some genetic groups (DAPC 6, 7, 9, 12) were not discriminated by SNP-derived markers. The phylogenetic relationship of these clusters to sublineages I and II remains to be determined, and should be clarified by additional genomic sequence analyses.

### MLVA-19 as part of a hierarchical Xvm typing system

While SNPs reveal little polymorphism, their phylogenetic signal is informative as it is not disturbed by homoplasic events. The little diversity obtained with SNP markers defined sub-lineages within Xvm but is not sufficient to track the strains of this genetically monomorphic pathogen during outbreaks [7]. We developed highly discriminatory TR markers suitable to separate Xvm isolates within populations which are congruent with SNP typing. In the future, both genotyping systems could be used together within a hierarchical typing procedure [16, 67], with the SNP markers being used to define the higher evolutionary groups at the lineage level, and our MLVA-19 scheme being used for outbreak investigations, regional surveillance, amount and directions of gene flows.

## Conclusion

We have established that the MLVA-19 scheme developed in this study is highly resolvent from the regional scale to the field scale. This genotyping tool is thus perfectly suited for exploring the genetic diversity of the recently emerging Xvm populations in East and Central Africa and could in future be helpful in addressing evolutionary and ecological questions that are important to address for deciphering the epidemiology of *Xanthomonas* wilt on banana, including the reconstruction of Xvm invasion routes throughout Africa. With the MLVA profiles deposited in MLVA Bank (http://bioinfo-web.mpl.ird.fr/MLVA_bank/Genotyping/), it will be possible to share data from new outbreaks or new emerging situations and compare them to the Xvm known genetic diversity for epidemiological investigations. This portable and simple genotyping tool can also be used in the future to assist the regional deployment of new Xvm-resistant banana and enset genitors.

## Supporting information

S1 Table TR Loci characteristics: Motif sequence, array homogeneity and completeness. ^a^The nucleotides differing from the reference TR sequence are written in bold. ^b^ Occurrence frequency of the alternative TR sequence.
(XLSX)

S2 Table MLVA-19 alleles in *X*.*vasicola* pv. *musacearum*: Summary of allele sizes and correspondence with raw number of repeats and rounded number of repeats. The alleles shared with other pathovars of *X*.*vasicola* are indicated in the column “shared with”. Xvv: *X*.*vasicola* pv. *vasculorum*; Xvh: *X*.*vasicola* pv. *holcicola*. ^a^ Raw repeat numbers were rounded to the next integer, following Pourcel [12].
(XLSX)

S3 Table Relative contributions (A) and correlations (B) of each variable (locus) to each of the first 5 dimensions of the PCA (R::FactoMineR::PCA). (XLSX)

S4 Table Composition of clonal complexes obtained from a collection of *Xanthomonas vasicola* pv. *musacearum* (n = 335) with their number of haplotypes and countries of origin.
(XLSX)

S5 Table Comparison of the haplotypes obtained from a core-collection of 36 Xvm strains using the MLVA-19 scheme and the two sets of SNP-derived RFLP markers and their discriminative Hunter-Gaston index. (XLSX)

S1 Fig Physical positions of the MLVA-19 loci on the chromosome of the Xvm complete genome NCPPB 4379. Loci are designated by their name, and their coordinates on the chromosome. The intragenic loci are framed.
(EPS)

S2 Fig Relationship between VNTR loci repeat sizes and locus polymorphism, as assessed by Nei’s gene diversity (A) and number of alleles (B). (EPS)

S3 Fig Principal Component Analysis (PCA) showing the correlation circle of each variable to the MLVA-19 scheme on the most informative axes 1 and 2. (EPS)

S4 Fig Principal Component Analysis (PCA) showing the correlation circle of each variable to the MLVA-19 scheme on the most informative axes 1 and 3. (EPS)
